# Influence of species composition and cultivation condition on peri-implant biofilm dysbiosis *in vitro*

**DOI:** 10.3389/froh.2025.1649419

**Published:** 2025-09-04

**Authors:** Nils Heine, Kristina Bittroff, Szymon P. Szafrański, Maya Duitscher, Wiebke Behrens, Clarissa Vollmer, Carina Mikolai, Nadine Kommerein, Nicolas Debener, Katharina Frings, Alexander Heisterkamp, Thomas Scheper, Maria L. Torres-Mapa, Janina Bahnemann, Meike Stiesch, Katharina Doll-Nikutta

**Affiliations:** 1 Department of Dental Prosthetics and Biomedical Materials Science, Hannover Medical School, Hannover, Germany; 2 Lower Saxony Center for Biomedical Technology, Implant Research and Development (NIFE), Hannover, Germany; 3 Institute of Technical Chemistry, Leibniz University Hannover, Hannover, Germany; 4 Institute of Quantum Optics, Leibniz University Hannover, Hannover, Germany; 5 Institute of Physics, University of Augsburg, Augsburg, Germany; 6 Centre for Advanced Analytics and Predictive Sciences (CAAPS), University of Augsburg, Augsburg, Germany

**Keywords:** dental plaque, dysbiosis, dental implants, microbiological techniques, dynamic cultivation

## Abstract

**Introduction:**

Changes in bacterial species composition within oral biofilms, known as biofilm dysbiosis, are associated with the development of severe oral diseases. To better understand this process and help establish early detection systems, models are needed which replicate oral biofilm dysbiosis *in vitro* – ideally by also mimicking natural salivary flow conditions.

**Methods:**

For this purpose, the present study cultivated two different combinations of oral commensal and pathogenic strains – *Streptococcus oralis, Actinomyces naeslundii, Veillonella dispar/parvula, Fusobacterium nucleatum* and *Porphyromonas gingivalis* – comparatively within an established flow chamber model on the implant material titanium, and statically in 6-well plates for 21 days. Biofilm morphology, species distribution, and bacterial metabolism were analyzed by fluorescence microscopy, molecular biological methods, and metabolic interaction prediction.

**Results:**

Biofilm growth and composition were strongly influenced by bacterial species selection, and to a more minor extent, by cultivation conditions. Within the model containing *V. dispar* and a laboratory *P. gingivalis* strain, a diversification of commensal species was observed over time along with a significantly reduced pH-value. In contrast, the model containing *V. parvula* and the clinical isolate *P. gingivalis* W83, a dysbiotic shift with increased pathogen levels, pH-value, and virulence factors was achieved.

**Conclusion:**

Within the present study, different *in vitro* oral multispecies biofilm models were successfully developed. Depending on bacterial species selection, these models were able to depict the infection-associated dysbiotic shift in species composition under flow conditions solely by intrinsic interactions and without the use of external stimuli.

## 1 Introduction

Bacterial biofilms of the oral cavity – also known as dental plaque – are associated with the development and progression of multiple oral diseases. These biofilms are formed by a multitude of oral bacterial species that adhere both to surfaces and to each other. These bacteria successfully protect themselves within an extracellular matrix, resulting in drastically increased tolerance towards the immune system and antibiotic treatment. In this regard, biofilm formation on dental implants in particular is closely linked to progressive diseases, since the implant lacks an innate immune response and other protective anatomical features (1). Peri-implant mucositis and peri-implantitis – analogous to gingivitis and periodontitis on natural teeth – are associated with severe inflammatory reactions that can lead to subsequent soft- and hard-tissue destruction. From the microbiological perspective, the progression of peri-implantitis/periodontitis is often accompanied by a notable shift in the biofilm species composition and activity (2). Whereas the initial commensal biofilm is dominated by mitis-group streptococci, *Actinomyces* and *Veillonella* species, advanced disease state biofilms frequently exhibit larger amounts of *Prevotella* and *Peptostreptococacae* species (2). This process of disease-associated changes in bacterial species composition is called bacterial dysbiosis.

To prevent the onset of peri-implantitis, early detection of bacterial dysbiosis would allow for a timely treatment that also circumvents tolerance development. However, the establishment of dysbiosis sensors – e.g., based on spectroscopy or chemometrics – requires reproducible *in vitro* models to serve as test systems. Oral multispecies biofilm *in vitro* models typically contain up to ten characteristic bacterial species that are either sampled from volunteers or commercially available type strains (3–5). These biofilms are grown on various materials (including implant-grade titanium) for several days or weeks under either static or salivary shear force-mimicking dynamic conditions (6, 7). One example is the Hannoverian Oral Multispecies Biofilm Implant Flow Chamber (HOBIC) model developed in our group, which contains the oral commensals *Streptococcus oralis*, *Actinomyces naeslundii*, *Veillonella dispar* as well as the oral pathogen *Porphyromonas gingivalis (*8). This four-species biofilm is grown on titanium discs in custom-made flow chambers designed for non-invasive microscopic readout. Within the incubation time of 24 h, reproducible biofilms of commensal composition are formed. In contrast, Siddiqui et al. have reported on a similar six-species biofilm model on titanium that was cultivated under static conditions for 21 days (6). Over time, a clear shift in bacterial species composition towards the increase of pathogenic species could be detected. However, this model lacks the naturally existing flow shear forces.

The aim of the present study was to advance the HOBIC model and reproduce the bacterial dysbiosis associated with peri-implantitis *in vitro*. For this purpose, two different five-species combinations were grown comparatively under both static and dynamic conditions over 21 days and analyzed for bacterial growth (optical density and pH development), biofilm morphology (live/dead fluorescence staining with confocal microscopy and digital image analysis) and species distribution (quantitative real-time PCR and fluorescence *in situ* hybridization). By this, the research hypotheses that (I) a bacterial shift can be introduced solely by increasing cultivation time as well as that (II) the shift depends on species composition and (III) cultivation conditions were addressed.

## 2 Materials and methods

### 2.1 Bacterial strains and culture conditions

Bacteria were routinely stored as glycerol stocks at −80°C. *Veillonella dispar* DSM 20735 (German Collection of Microorganisms and Cell Cultures GmbH, DSMZ, Braunschweig, Germany), *Veillonella parvula* ATCC® 17745™ (American Type Culture Collection, ATCC, Manassas, VA, USA), *Fusobacterium nucleatum* DSM 15643, *Porphyromonas gingivalis* DSM 20709 and *Porphyromonas gingivalis* ATCC W83, were streaked out on fastidious anaerobe agar (Lab M Ltd., Heywood, UK) plates supplemented with 5% defibrinated sheep blood (Thermo Fisher Scientific Inc., Waltham, MA, USA) and incubated at 37°C under anaerobic conditions, which were achieved using AnaeroGen™ bags (Thermo Fisher Scientific Inc.), for three days. Afterwards, colonies from the agar plates were transferred to liquid medium and cultured overnight in brain heart infusion medium (BHI, Oxoid Deutschland GmbH, Wesel, Germany) supplemented with 10 mg/L vitamin K (Carl Roth GmbH + Co. KG, Karlsruhe, Germany) (BHI + VitK, *V. dispar/parvula*) or in fastidious anaerobe broth (Lab M Ltd., *F. nucleatum* and *P. gingivalis*) at 37°C under anaerobic conditions. *Streptococcus oralis* ATCC 9811™ and *Actinomyces naeslundii* DSM 43013 were cultured overnight in BHI + VitK at 37°C under anaerobic conditions.

### 2.2 Static biofilm growth in well plates

Bacterial overnight cultures were adjusted to an optical density at 600 nm (OD_600_) of 0.05 in BHI + VitK with 5 mg/L hemin (BHI + VitK/Hem, Sigma Aldrich, St. Louis, MO, USA), and mixed in two different five-species combinations: *S. oralis*, *A. naeslundii* and *F. nucleatum* were combined either with *V. dispar* and *P gingivalis* DSM 20709 (commensal model) or *V. parvula* and *P. gingivalis* ATCC W83 (dysbiotic model). 5 ml per well of the mixed suspension were directly added to polystyrene 6-well plates and incubated for 1, 3, 6, 10, 15, or 21 days at 37°C under anaerobic conditions. Every other day, half of the medium was replaced with fresh medium. Before analysis, biofilms were washed once with phosphate buffered saline (PBS, Sigma Aldrich).

### 2.3 Biofilm growth in the adaptive HOBIC model

**Figure 1:**
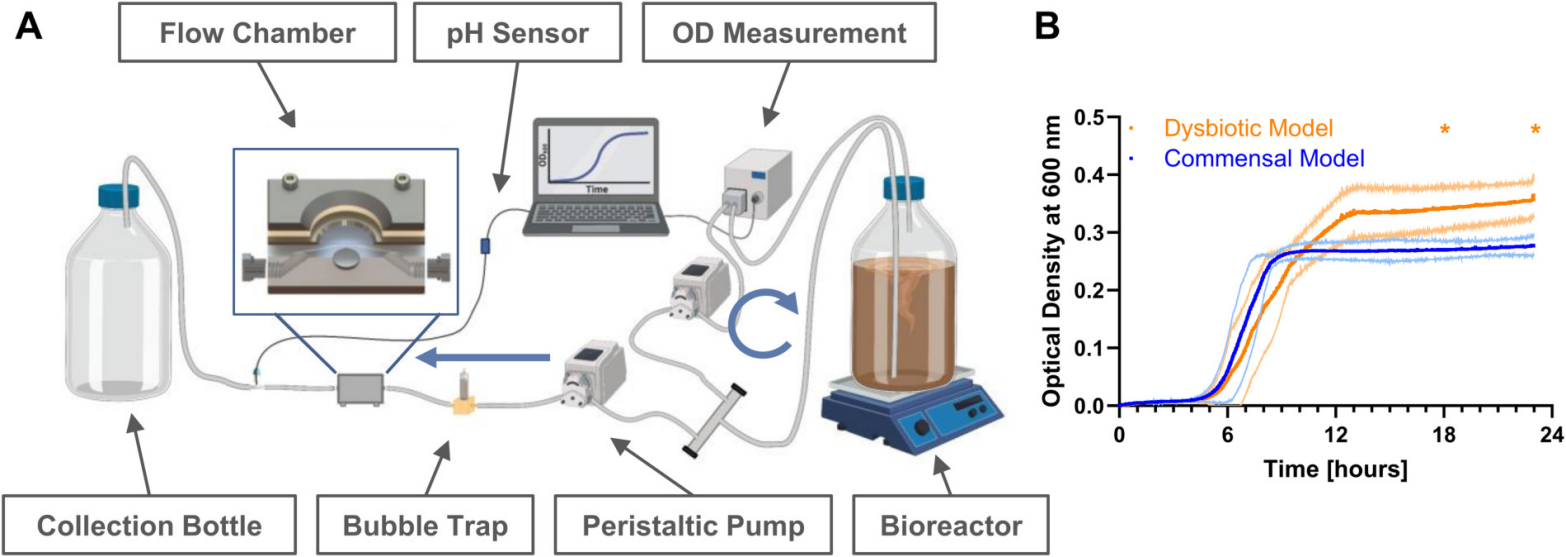
Bacterial growth in the hannoverian oral multispecies biofilm implant flow chamber (HOBIC) model. **(A)** Schematics of the flow chamber system with indicated components. Blue arrows indicate flow direction with 100 µl/min. Created with BioRender, Winkel. A. (2025) https://BioRender.com/ d73y389. **(B)** Bacterial growth curves (mean ± standard deviation) of commensal and dysbiotic species composition within the first 24 h in the bioreactor measured by an inline photometer at *λ* = 600 nm. Significant differences (*) between the commensal and dysbiotic model after 18 and 24 h could be identified with *p* ≤ 0.05 (*N* = 5).

The setup of the flow chamber system is shown in Figure 1A, and is based on the previously described “Hannoverian Oral Multispecies Biofilm Implant Flow Chamber (HOBIC)” model (8) with the following modifications: In this case, pH-sensitive flow-through cells connected to optical fibers (FTC-SU-LG1-S, PreSense Precision Sensing GmbH, Regensburg, Germany) were integrated behind the flow chambers. Grade 4 titanium discs (12 mm diameter, 1.5 mm height, R_a_ = 0.31 µm) were submerged in artificial saliva (850 mg/L mucin, 10 µg/ml lysozyme, 1 mg/ml α-amylase, 40 µg/ml albumin) during sterile assembly of the chambers. Then, the chambers were integrated into the system and 2.1 ml per strain with OD_600_ of 0.05 were added to 1.5 L BHI + VitK/Hem in the bioreactor. In addition to the previously described HOBIC model (8), *F. nucleatum* was added as fifth bacterium to the inoculum for both combinations and *V. dispar* and *P. gingivalis_20709_* were replaced by *V. parvula* and *P. gingivalis_W83_* as “dysbiotic” strain combination. After 24 h of cultivation with 100 µl/min at 37°C under anaerobic conditions, the components up to the bubble trap (Figure 1A) were replaced with new sterile parts, except for the OD measuring bypass. The system was then run for 20 additional days with sterile 1:2 diluted medium. On day 1, 3, 6, 10, 15, and 21, chambers were washed with PBS for 30 min at 100 µl/min before subjected to further analysis. Optical density of the first 24 h and pH development were recorded with *N* = 5 replicates per condition. Statistical comparisons of each parameter between the models at individual time points were done using 2-way ANOVA with Śidák's correction for multiple comparison.

### 2.4 Live/dead staining and microscopic analysis

Static and flow chamber biofilms were stained using SYTO®9 and propidium iodide (PI) of the LIVE/DEAD® BacLight™ Bacterial Viability Kit (Life Technologies, Darmstadt, Germany) at a concentration of 1:2000 of the stock solutions in PBS, followed by fixation with 2.5% glutardialdehyde. For staining of flow chamber biofilms, the dye and fixation solutions were pumped through the system as previously described (8). Likewise, confocal laser-scanning microscopy was done using established protocols (8). For the HOBIC systems, from day 6 onwards, images were taken from the cover slip downwards rather than directly on the titanium surface, since the laser could not reach through the thick biofilm. From at least *N* = 15 images per condition, biofilm volume and live/dead distribution were quantified using the software Imaris (v8.4.1, Bitplane AG, Zurich, Switzerland). For statistical analysis, biofilm volume data were tested for normal distribution using D'Agostino & Pearson Omnibus Normality test followed by Kruskal–Wallis test with Dunn's multiple comparison correction. Biofilm viability data were tested using 2-way ANOVA with Tukey's multiple comparison test.

### 2.5 PMA treatment, DNA extraction, and qRT-PCR

After microscopy, the biofilm inside the chambers was harvested and either directly frozen or subjected to additional PMA treatment (9). Following washing with PBS, bacteria were incubated with 0.2 mM PMAxx™ (Biotium, Inc., Fremont, CA, USA) for 10 min at 4°C, and then again for 20 min in the PMA-Lite™ LED Photolysis Device (Biotium, Inc.). DNA was extracted using a customized protocol which deploys a combination of enzymatic lysis, mechanical disruption, and column-based DNA isolation. Bacterial sample material was initially treated with 450 µl lysozyme solution [20 mg/ml lysozyme (Merck, Darmstadt, Germany) in 20 mM Tris HCl, pH 8.0; 2 mM EDTA; 1,2% Triton] for two hours at 37°C. After addition of 50 µl Proteinase K and 500 µl AL buffer (both Qiagen, Hilden, Germany), treatment was extended for 30 min at 56°C and 15 min at 95°C. The complete sample material was then transferred to Lysing Matrix E tubes (MP Biomedicals, Eschwege, Germany) and mechanically disrupted in three cycles of 6,500 rpm for 30 s in a Precellys 24 homogenizer (Bertin Technologies, Frankfurt am Main, Germany), punctuated with cooling on ice for five minutes in between cycles. Finally, beads and debris were sedimented by centrifugation (5 min, 14,000 × g), and the cleared supernatant was mixed 1:1 with 100% ethanol. Subsequent steps were performed with the QIAamp Mini Kit (Qiagen) according to the manufacturer's protocol “DNA Purification from Blood or Body Fluids” – starting with the application to the spin columns. To reduce the risk of contamination, a new collection tube was used after each of the kit-specific wash steps. DNA was eluted with 50 µl of PCR-grade water and stored at −20°C until further usage.

Quantitative real-time PCR and calculation of respective cell numbers and relative species distribution were performed as described by Kommerein et al. (9) using the SYBR Green reaction mix (Bio-Rad Laboratories GmbH, Feldkirchen, Germany) and the LightCycler 96 (Roche Holding GmbH, Grenzach-Wyhlen, Germany) with *N* = 9 replicates per condition. Primer pairs, reaction components, cycle conditions and genome weight per cell are all given in the Supplementary Tables S1–S4, respectively. Statistical comparisons for individual species development over time was done using 2-way ANOVA with Dunnett's test for multiple comparison.

### 2.6 Fluorescence-*In-situ*-hybridization

To prepare HOBIC samples for representative fluorescence *in situ* hybridization (FISH), 50% (v/v) ethanol was pumped through the system for 20 min with a flow rate of 250 μl/min, after which the ethanol filled chambers were removed from the system and then stored at 4°C overnight to fixate the bacteria. The chambers were then opened, and the titanium specimen were transferred to a 6-well plate and left to dry under sterile conditions. FISH staining and CLSM analysis were performed as previously reported (8, 10). Briefly, 1 g/L lysozyme (Merck) treatment at 37°C for 10 min was used to disrupt cell membranes. Lysis was stopped with pure ethanol, samples were dried and then stained with six fluorescently labeled 16S rRNA probes (Supplementary Table S5) in hybridization buffer at 46°C for 30 min. *F. nucleatum* was targeted by two probes that shared the same nucleotide sequence, but were labeled with different dyes – resulting in co-localized blue and red fluorescence. Afterwards, samples were washed several times and analyzed by CLSM. A 630-fold magnification was used to take image stacks with an xy-size of 185 × 185 µm^2^ and a 2 µm z-step-size. Scanning was done sequentially per frame. The first sequence used a 405 nm and a 552 nm laser for excitation, and detected blue and yellow signals in the wavelength ranges 413–477 nm and 576–648 nm, respectively. During the second sequence, a 488 nm and a 638 nm laser were used to excite the samples, and emission detection was done in the wavelength ranges 509–576 nm and 648–777 nm for green and red signals, respectively.

### 2.7 Gingipain-specific enzyme-linked immunosorbent assay (ELISA)

Supernatants of the HOBIC model flow chambers were collected, frozen, and used for gingipain protein quantification using the human *P. gingivalis-*specific IgG antibody ELISA kit (Huangshi INS Biological Technology Co., Ltd., Huangshi, China). Analysis of *N* = 9 replicates was done according to the manufacturer's protocol – but with the samples being additionally incubated for 1 h at room temperature followed by 3 washing steps before addition of the detection antibody. Statistical comparisons of gingipain concentration between the models at individual time points was done using 2-way ANOVA with Śidák's correction for multiple comparison.

### 2.8 Literature-based metabolic interaction prediction

The potential of biofilm members to engage in metabolic and enzyme-based interspecies interactions was inferred using a custom database (11). Interaction data were sourced from the literature and subjected to manual curation (12, 13). Custom-designed graphs were employed to visualize the interaction networks.

### 2.9 Statistical analysis

Data presentation and statistical analysis were done using GraphPad Prism 8.4 (GraphPad Software Inc., San Diego, CA, USA). Statistical test details can be found in the respective methods section. Family-wise significance level was defined with *α* = 0.05.

## 3 Results

### 3.1 Time-dependent biofilm growth and viability

**Figure 2:**
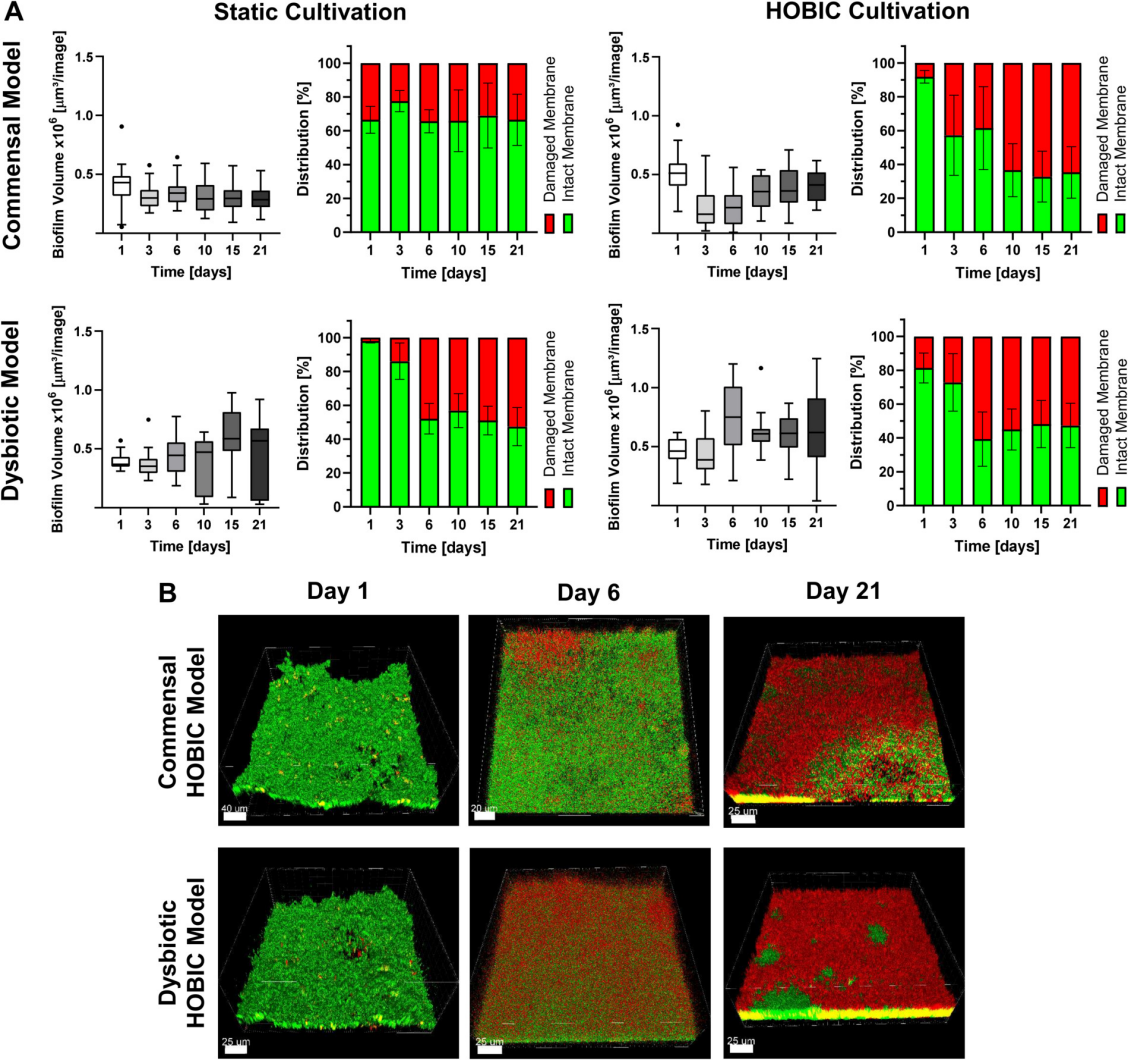
Biofilm volume and viability development over time of the different oral multispecies models. **(A)** Tukey box plots of biofilm volume per image (each left) and mean ± standard deviation of membrane-based biofilm viability (each right) of the commensal and dysbiotic models during static and HOBIC cultivation over time analyzed by fluorescence staining and CLSM. Results of statistical evaluation are given in Supplementary Tables S6, S7. **(B)** Representative 3D reconstructed CLSM images of the commensal and dysbiotic HOBIC model at different time points. Green fluorescence indicates viable cells with intact membrane, whereas red/yellow fluorescence indicates cells with damaged membrane.

Initial bacterial growth in the bioreactor was monitored using inline optical density measurement, and showed typical bacterial growth curves with significantly increased growth of the dysbiotic model (Figures 1A,B). Subsequent biofilm growth on titanium discs was then analyzed by fluorescence staining and confocal microscopy. Within both commensal models, the biofilm volume significantly decreased after one day, and then re-established until day 10 (Figure 2A, Supplementary Table S6). This development was more pronounced in the commensal HOBIC model. In contrast, the biofilm volume of both dysbiotic models significantly increased after day 1, reaching a plateau at day 6 (HOBIC system) and day 15 (static system), respectively (Figure 2A, Supplementary Table S6). Biofilm viability – analyzed by fluorescence-based membrane integrity – was observed to significantly decrease over time for all cultivation conditions except for the static commensal model (Figures 2A,B, Supplementary Table S7). Viability development thereby replicates the respective biofilm volume pattern.

### 3.2 Cultivation condition-dependent species composition

**Figure 3:**
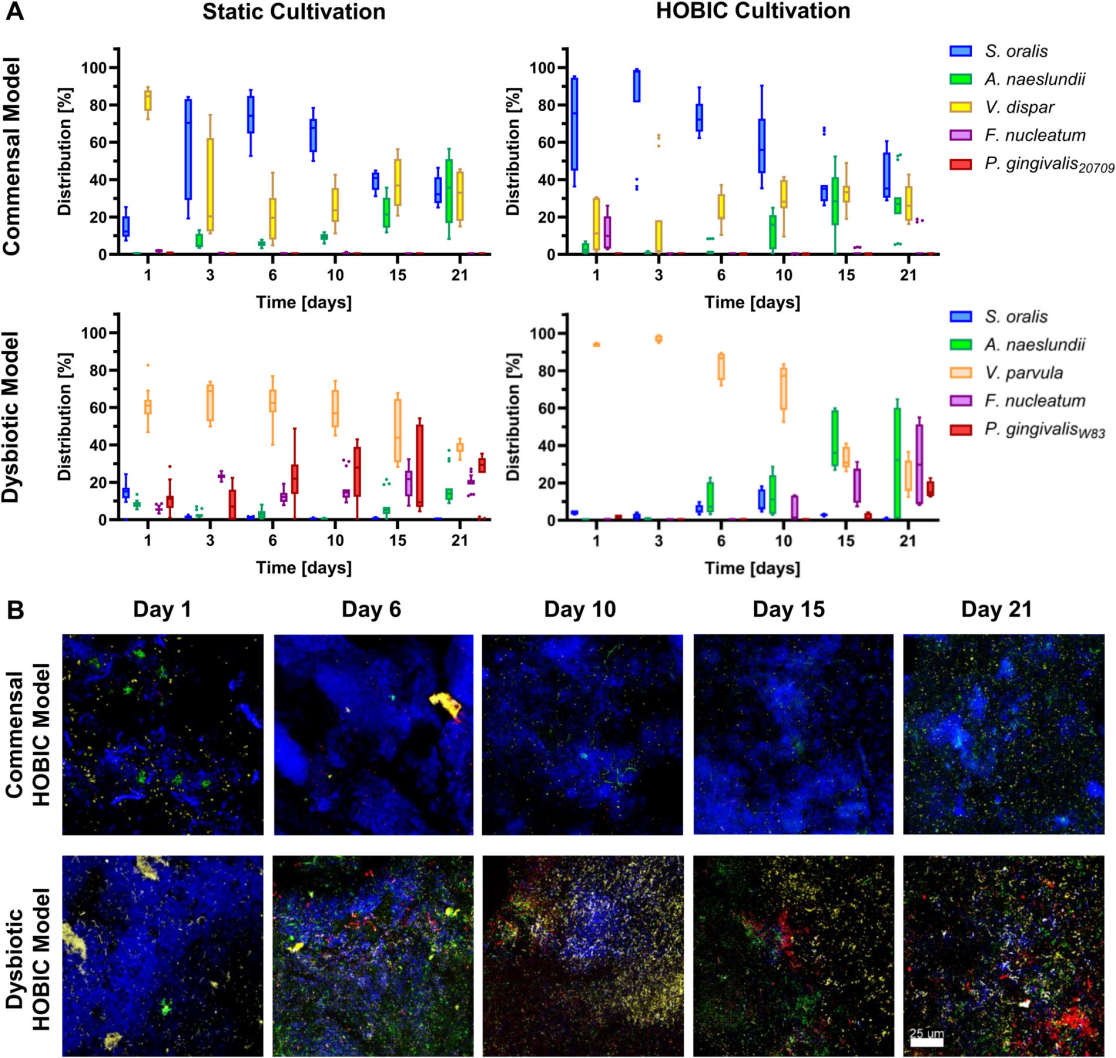
Viable bacterial species distribution over time of the different oral multispecies biofilm models. **(A)** Tukey box plots of individual species distributions of the commensal and dysbiotic models during static and HOBIC cultivation over time quantified by qRT-PCR with PMA pre-treatment. Results of statistical evaluation are given in Supplementary Table S8. **(B)** Representative FISH images of the commensal and dysbiotic HOBIC model at different time points. Color coding is similar to those of **(A)**.

Time-dependent species composition (both, viable and total count) was analyzed by DNA isolation and qRT-PCR as well as FISH staining, and these analyses revealed clear differences between the commensal and dysbiotic model (Figures 3A,B, Supplementary Figure S1). The changes in bacterial species distribution were more pronounced for viable cells (Figure 3) than for the total count (Supplementary Figure S1). Within the commensal HOBIC model, viable *S. oralis* was the initially dominant species, although its amount significantly decreased over time from approx. 70% to merely 35% (Supplementary Table S8). Within the commensal static model, this decrease was also observed – but only from day 6. Initially, *V. dispar* was the dominant species, but was then gradually replaced by *S. oralis* up until day 6. Afterwards, *V. dispar*'s distribution remained on average stable at 30% within both commensal models. In parallel, *A. naeslundii* established itself with prolonged incubation to approx. 30%. *F. nucelatum* and *P. gingivalis* were almost undetectable in both commensal models. In contrast, total and viable species distribution of the dysbiotic model differed remarkably (Figures 3A,B, Supplementary Figure S1). For the static system, *V. parvula* remained the dominant species independent of incubation time (50%–60%), followed by *P. gingivalis* (25%), *F. nucleatum* (20%), and only a very low amount *S. oralis* and *A. naeslundii*. In the HOBIC system, the initially dominant *V. parvula* significantly decreased from approx. 95% to 20% (Supplementary Table S8), while *A. naeslundii*, *F. nucleatum* and *P. gingivalis* successively increased from day 6, 10, and 21, respectively, up to 15%–30% (Figure 3B).

### 3.3 Species composition-dependent biofilm metabolism

**Figure 4:**
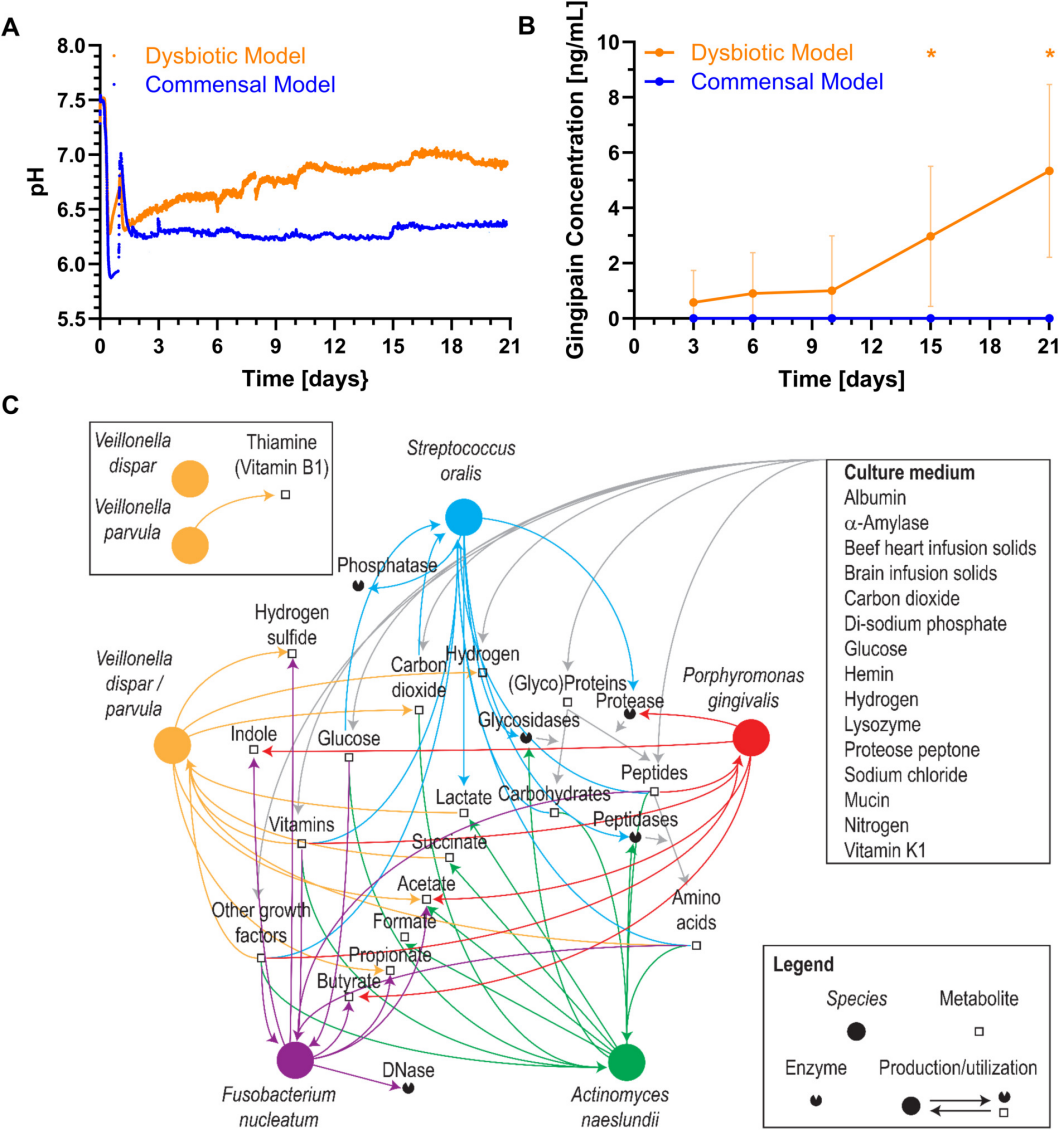
Bacterial metabolism of the different oral multispecies biofilm models. Mean ± standard deviation of **(A)** pH values and **(B)** gingipain protein concentrations over time in the commensal and dysbiotic HOBIC models. The peak in pH value after day 1 results from the change to fresh, sterile medium. Statistically significant differences with *p* ≤ 0.05 are marked with (*) (*N* = 5 for pH, *N* = 9 for gingipain). **(C)** Potential metabolic interactions between the different bacterial species interfered using a custom-made database (11) summarizing curated phenotypic information (12, 13). Nodes representing species were placed on an arbitrary circle.

Within the commensal and dysbiotic HOBIC models, pH-values and *P. gingivalis* gingipain protein concentration over time were determined by optical fiber measurement and ELISA, respectively, and showed clear differences (Figures 4A,B). For the commensal model, pH-values initially dropped below pH 6.0, sharply increased upon medium change at day 1, and then established itself at approx. pH 6.3. In contrast, for the dysbiotic model, pH-values only dropped to pH 6.3 and then gradually increased to pH 6.9 by day 21. These higher pH-values showed a tendency to explicitly increase the growth of the dysbiotic model's *P. gingivalis* strain (Supplementary Figure S2A). In line with these observations, the amount of gingipain protein significantly increased over time only within the dysbiotic model (Figure 4B). To account for these differences, literature-based prediction of metabolic interactions between the six bacterial species was performed (Figure 4C). Based on the available nutrients from the culture medium, the species could engage in multiple food chain and enzyme sharing behaviors, with peptides, glucose, vitamins and other growth factors produced by *S. oralis*, *A. naeslundii* and *V. dispar/parvula* and then utilized by *F. nucleatum* and *P. gingivalis*. Whereas for *V. dispar* and *V. parvula* generally similar metabolic pathways could be found, only *V. parvula* could have the capability of *de novo* thiamine (vitamin B1) synthesis. However, although the dysbiotic model's *P. gingivalis* strain showed increased overall growth compared to the commensal model's strain, no growth difference in Veillonella-preconditioned medium could be detected (Supplementary Figure S2B).

## 4 Discussion

Early detection of oral biofilm dysbiosis on dental implants can prevent the development of severe infections like peri-implantitis. To help establish dysbiosis sensors, however, reliable *in vitro* models must first be developed for use as test systems. Within the present study, the existing HOBIC model was successfully adapted to reproduce bacterial dysbiosis for this purpose. With regard to the initial research hypotheses, this model helped to confirm that the dysbiotic shift depended both on the selected bacterial species as well as on the cultivation conditions.

The bacterial characteristic oral species selected for this study were *S. oralis, A. naeslundii, V. dispar* or *V. parvula, F. nucleatum,* and *P. gingivalis.* All genera but *Fusobacterium* were already included in the previous HOBIC model (8, 9). *S. oralis* and *A. naeslundii* are among the dominant primary oral colonizers associated with oral health (2). *V. dispar* and *V. parvula* are part of the core microbiome, co-aggregate with *S. oralis* and *A. naeslundii*, and metabolize lactate produced by them (2, 14). *P. gingivalis* is part of the red complex bacteria and associated with periodontitis and peri-implantitis (2). As the biofilm of the initial HOBIC model mainly consisted of *S. oralis, A. naeslundii*, and *V. dispar*, and contained only a small proportion of *P. gingivalis*, it depicts an early commensal oral biofilm (8, 9). To support the dysbiotic shift, *F. nucleatum* was added as fifth bacterium in this study. The species is considered a central bridging bacterium between commensal and dysbiotic strains as it co-aggregates with bacteria of both groups (2). The species selection closely matched those of other commensal and dysbiotic oral biofilm models (5–7, 15). Blank et al., Sanchez et al. and Siddiqui et al. used *S. oralis, A. naeslundii, V. parvula, F. nucleatum, P. gingivalis,* and, in contrast to this study, additionally *Aggregatibacter actinomycetemcomitans* for their dysbiotic/pathogenic biofilm models (5–7). Zhang et al. combined *Streptococcus mitis, A. naeslundii, V. parvula, F. nucleatum,* and *Campylobacter gracilis* with *P. gingivalis* or *Prevotella intermedia* to obtain commensal and pathogenic biofilm compositions (15). Therefore, all models integrated species from different Socransky complexes (16), with similar species of the “red” (*P. gingivalis*) and “orange” (*F. nucleatum*) complexes associated with periodontitis, and slight variation in the selection of species from complexes associated with periodontal health. However, currently several hundred species have been identified in the oral cavity that synergistically and antagonistically interact with each other (2). Thus, it bears noting that all models remain simplifications of the natural microbiome complexity. All results generated by these *in vitro* models – including their interspecies interactions as well as interactions with the underlying surface and the surrounding – should therefore be interpreted with this limitation in mind and need to be verified by *in vivo* studies before (clinical) application. In addition, the presented bacterial dysbiosis model does not include any clinically relevant interactions with human cells, most importantly immune cells secreting antibacterial defensins. Recent *in vitro* studies have shown that the interaction of bacteria and human cells can drastically influence the effects of antibacterial substances (17, 18). To take this important aspect into account, cellular influence within the presented biofilm model should be studied by incubation with preconditioned supernatant or direct bacteria-cell co-culture in the future.

Biofilm growth of the selected bacteria was done in full medium supplemented with vitamin K and hemin with all bacterial species inoculated at the same time. These conditions are similar to several other dysbiosis/pathogenic biofilm models (5–7, 19) and support the growth of the pathogenic species *P. gingivalis* as identified in preliminary experiments. Within this experimental setup, the dysbiotic shift was induced by intrinsic bacterial interactions only, closely replicating natural oral conditions. In contrast, in the biofilm models of Dalwai et al. and Thurnheer et al., dysbiosis was induced by changing cultivation medium (increasing the volume of serum in modified saliva medium or artificial crevicular fluid) and reducing oxygen concentration alongside with inoculating bacterial species sequentially from commensals to pathogens (4, 20). Whereas these approaches offer the possibility of external control and analysis of the effect of each individual change, the setup selected here is inherently more beneficial for observing intrinsic processes – a pre-requirement for the development of dysbiosis sensors.

Biofilms in the HOBIC model were cultivated on saliva pre-conditioned titanium surfaces, whereas static biofilms were directly cultivated on polystyrene well plates. Most other biofilm models used saliva-coated hydroxyapatite as substratum, while Siddiqui et al. used titanium and zirconium without conditioning and Dalwai et al. used saliva-coated polystyrene plates (3–7, 15). Although the underlying surface significantly influences the initial bacterial adhesion, the effect on multilayered biofilm formation is typically considered lower. For example, whereas saliva coating influenced the first seconds of *S. oralis* adhesion forces on hydroxyapatite and resin surfaces, an effect on prolonged bacterial adhesion could not always be detected (21–23). In addition, the established four-species HOBIC model has already shown similar species distributions for growth on polystyrene, titanium and glass surfaces (8, 9, 24). Changes in biofilm composition are, thus, most probably not related to the material substrate. However, there is at least a limited number of studies that showed a dependency of healthy and pathogenic species distribution on the underlying topography (25, 26). Therefore, the difference in this parameter has to be kept in mind when comparing the results of the static and HOBIC model in the following and it should be addressed in further studies.

Over the incubation time of 21 days, biofilm volume increased for the dysbiotic model only, reaching a plateau after 6 days. This growth pattern has also been observed for other models with a similar dysbiotic species composition and cultivation time of more than 7 days, as well as for *in situ* grown biofilms on implant healing abutments (3, 6, 11). In comparison to the commensal model, the initial planktonic growth in the bioreactor of the HOBIC model, and the growth of *V. parvula* and *P. gingivalis_W83_* at different pH (Supplementary Figure S2A) was found to be significantly higher. This makes the increased growth of these individual species the most likely reason for the elevated biofilm volume of the dysbiotic model. The parallel decrease of biofilm viability independently of the species composition is also in line with previous *in vitro* and *in situ* results (5, 7, 11), and most likely due to limited nutrient availability in deeper layers of the maturated biofilm.

The most obvious difference between the commensal and dysbiotic models lies in their divergent species composition. During the first days, the commensal model was dominated by *S. oralis* after a short initial establishing phase. In contrast, the dysbiotic model was initially dominated by *V. parvula*. Differing proportions of *S. oralis* and *V. parvula* have already been described in previous oral biofilm models – with studies showing an initial dominance of *S. oralis* (5), an initial dominance of *V. parvula* (7) or equal amounts of both species (3, 6). Their establishment is probably significantly influenced by the (pre-culture) cultivation conditions; however, the details of these alterations remain to be analyzed in future studies. The different proportions of *S. oralis* are also the most probable reason for the differences in pH-values measured in the HOBIC model. As indicated in the predicted metabolic interactions and known from literature (27), *S. oralis* utilizes carbohydrates from the medium to produce lactate via fermentation, causing strong medium acidification. In contrast, Veillonellaceae metabolize nutrients (including lactate) to less acidic acetate and propionate (28), resulting in higher pH-values in the medium.

With prolonged cultivation time, a diversification of commensal species was observed in the commensal model. In contrast, the dysbiotic model showed a notable increase in pathogenic species with reduced proportions of commensal strains. This observation is further supported by the increase in gingipain proteins, which are trypsin-like cysteine proteases that are among the most important *P*. *gingivalis* virulence factors (29). Since these models only differed in the *Veillonella* species and the *P. gingivalis* strain, their contribution seems to be crucial for the dysbiotic shift – at least within the limited setting of an *in vitro* experiment. Further evidence to support this observation can also be found in the literature: In a co-association study of *Streptococcus mutans, Veillonella dispar* and *Veillonella parvula* within the context of root caries, only *V. parvula* was found to support *S. mutans* growth *in vitro* (30)*.* More importantly, a metatranscriptomic analysis of different oral *in vitro* biofilm models found that *P. gingivalis_W83_* – a virulent strain which was also used for the dysbiotic model of this study – had a greater effect on biofilm dysbiosis than a lower virulent type strain by specifically influencing genes related to metabolic pathways and quorum sensing of several commensal species (15). An effect on the strain level would also be supported by the species-level metabolic interaction prediction of this study, where only a minor difference in thiamine (vitamin B1) production solely by *V. parvula* could be identified. Even though *P. gingivalis* could utilize vitamins produced by Veillonellaceae (31), they exhibit the enzymes for thiamine metabolism themselves according to KEGG-pathway analysis, and thus probably would not rely on cross-feeding by *V. parvula*. On the other hand, although *P. gingivalis_W83_* showed increased growth compared to *P. gingivalis_20709_*, Veillonella-conditioned medium had no different effect on both strains. In summary, these results strongly underscore the importance of further analyzing metabolic interaction within oral microbial communities that seem to significantly contribute to dysbiosis development within basic research approaches. The models developed here can be used for initial insights as well as validation for this purpose.

Aside from strain selection, cultivation conditions also influenced bacterial species composition – albeit to a more minor extent. Previous oral biofilm dysbiosis models have been primarily conducted under static conditions, with only the model of Dalwai et al. being conducted in a bioreactor but with bacterial species being added sequentially (3–6). A direct comparison of static vs. dynamic cultivation conditions of the same biofilm model has not been conducted so far. For both the commensal and dysbiotic model of this study, changes in species composition were more pronounced in the dynamic HOBIC than the static system. During static cultivation (even though medium was exchanged every other day), metabolites accumulated within the biofilm, probably making metabolite-based changes of species composition slower or less pronounced. In contrast, during cultivation in the HOBIC system, medium is constantly replaced, thus, preventing the metabolites from accumulating to a greater degree which might very well induce changes in species composition.

## 5 Conclusion

Within the present study, different *in vitro* oral multispecies biofilm models were successfully developed. Depending on bacterial species selection, these models were able to depict the infection-associated dysbiotic shift in species composition solely by intrinsic interactions, and without any deployment of or reference to external stimuli. The different results between cultivation conditions offer the possibility for a number of different future application: For the direct comparison between commensal and dysbiotic biofilms, straightforward static cultivation can be used with species composition being already different after 24 h. In contrast, for the observation of the bacterial shift over time (for example by novel sensor systems), the dysbiotic HOBIC model is to be preferred. The results of this study also point towards the (current) limitations of *in vitro* models and the need for further *in vivo* studies that focus on examining how metabolic interactions on the strain level influence bacterial species composition. For the validation of these *in vivo* observations, we believe that the presented biofilm models will serve as a valuable tool.

## Data Availability

The raw data supporting the conclusions of this article will be made available by the authors, without undue reservation.
