# Supplementary material for: Influence of species composition and cultivation condition on peri-implant biofilm dysbiosis in vitro
Source: Front Oral Health. 2025 Sep 4;6:1649419. doi: 10.3389/froh.2025.1649419 (PMC12443849; doi:10.3389/froh.2025.1649419)
Supplement: Supplementary file 1 [file Datasheet1.pdf]

Supplementary Material

Influence of species composition and cultivation condition on peri-implant biofilm dysbiosis *in vitro*

Nils Heine[1,5], Kristina Bittroff[1,5], Szymon P. Szafrański[1,5], Maya Duitscher[1,5], Wiebke Behrens[1,5], Clarissa Vollmer[1,5], Carina Mikolai[1,5], Nadine Kommerein[1,5], Nicolas Debener[2], Katharina Frings[3,5], Alexander Heisterkamp[3,5], Thomas Scheper[2,5], Maria L. Torres-Mapa[3,5], Janina Bahnemann[4,6], Meike Stiesch[1,5,†], Katharina Doll-Nikutta[1,5,†,*]

[1]Department of Dental Prosthetics and Biomedical Materials Science, Hannover Medical School, Hannover, Germany

[2]Institute of Technical Chemistry, Leibniz University Hannover, Hannover, Germany

[3]Institute of Quantum Optics, Leibniz University Hannover, Hannover, Germany

[4]Institute of Physics, University of Augsburg, Augsburg, Germany

[5]Lower Saxony Center for Biomedical Technology, Implant Research and Development (NIFE), Hannover, Germany

[6]Centre for Advanced Analytics and Predictive Sciences (CAAPS), University of Augsburg, Augsburg, Germany

[†]Equally contributing last authors

*** Correspondence:**
Katharina Doll-Nikutta
Nikutta.Katharina@mh-hannover.de

Table S1. Primer pairs used for qRT-PCR.

Species	Sequence	Gene	Reference
S. oralis	F: 5´-TCC-CGG-TCA-GCA-ACC-TCC-AGC-C-3´	*gtfR*	(Hoshino et al., 2004)
	R: 5´-GCA-ACC-TTT-GGA-TTT-GCA-AC-3´		
A. naeslundii	F: 5´-CAA-CGT-CGA-GGA-GAT-CCA-GG-3´	*gyrA*	(Kommerein et al., 2017)
	R: 5´-TAT-TGA-GGA-CCT-TGG-CG-3´		
V. dispar/ V. parvula	F: 5´-TGG-AGC-AAA-CCC-GAG-AAA-CA-3´	*16S rRNA*	(Kommerein et al., 2017)
	R: 5´-TTC-ACC-GCA-GTA-TGC-TGA-CC-3´		
F. nucleatum	F: 5´-CGC-CCG-TCA-CAC-CAC-GAG-A-3´	*16S rRNA*	this study
	R: 5´-ACA-CCC-TCG-GAA-CAT-CCC-TCC-TTA-C-3´		
P. gingivalis	F: 5´-AGG-CAG-CTT-GCC-ATA-CTG-CG-3´	*16S rRNA*	(Ashimoto et al., 1996)
	R: 5´-ACT-GTT-AGC-AAC-TAC-CGA-TGT-3´		

Table S2. Reaction components for a single qRT-PCR.

Reagent	Volume	Concentration
SYBR Green	12,5	1x
Forward Primer	0,5	0.2 µM
Reverse Primer	0,5	0.2 µM
Water, PCR grade (Roche Holding GmbH, Grenzach-Wyhlen, Germany	variable	-
Template DNA	variable (1-40 ng)	40 pg – 1.6 ng

Table S3. Cycle conditions for qRT-PCR.

Step	Temperature [°C]	Duration [sec]	Cycles
Pre-Denaturation	95	180	1x
Denaturation	95	10	40x
Annealing	56 (*P. gingivalis*)<br>58 (*S. oralis, A. naeslundii, V. dispar/parvula*)<br>60 (*F. nucleatum*)	20	
Elongation	72	20	
Post-Elongation	60	6	115x

Table S4. Genome size and genome weight used to convert qRT-PCR results into cell numbers.

Species	Genome size [bp]	Genome weight [ng]
S. oralis	1.96×10^{6}	2.15×10^{-5}
A. naeslundii	3.04×10^{6}	3.33×10^{-5}
V. dispar	2.12×10^{6}	2.32×10^{-6}
V. parvula	2.16×10^{6}	2.37×10^{-6}
F. nucleatum	2.17×10^{6}	2.38×10^{-6}
P. gingivalis	2.34×10^{6}	2.57×10^{-6}

Table S5. 16S rRNA FISH probes

Species	Name	Sequence	Label
S. oralis	So405	ACA gCC TTT AAC TTC AgA CTT ATC TAA	Alexa Fluor 405
A. naeslundii	An488	Cgg TTA TCC AgA AgA Agg gg	Alexa Fluor 488
V. dispar/<br>*V. parvula*	Vd568	AAT CCC CTC CTT CAg TgA	Alexa Fluor 568
P. gingivalis	Pg647	CAA TAC TCg TAT CgC CCg TTA TTC	Alexa Fluor 647
F. nucleatum	FUS664-blau	CTT gTA gTT CCg CYT ACC TC	Alexa Fluor 405
F. nucleatum	FUS664-rot	CTT gTA gTT CCg CYT ACC TC	Alexa Fluor 647

Table S6. Statistical analysis results (adjusted p-values) of **biofilm volume** comparisons over time. Data (at least N = 15 individual images per condition), were tested for normal distribution using D'Agostino & Pearson Omnibus Normality test followed by Kruskal-Wallis test with Dunn's multiple comparison correction. Family-wise significance level was set to α = 0.05 and statistically significant differences are highlighted in bold.

Static Cultivation

Commensal Model

	1	3	6	10	15	21
1		**0.005**	0.362	**0.004**	**0.002**	**0.001**
3			>0.999	>0.999	>0.999	>0.999
6				>0.999	>0.999	0.870
10					>0.999	>0.999
15						>0.999
21						

Dysbiotic Model

	1	3	6	10	15	21
1		>0.999	>0.999	>0.999	**0.003**	>0.999
3			>0.999	>0.999	**<0.001**	0.367
6				>0.999	0.151	>0.999
10					**0.039**	>0.999
15						0.750
21						

HOBIC Cultivation

Commensal Model

	1	3	6	10	15	21
1		**<0.001**	**<0.001**	**0.042**	0.394	0.936
3			>0.999	0.507	0.076	**0.027**
6				0.670	0.107	**0.039**
10					>0.999	>0.999
15						>0.999
21						

Dysbiotic Model

	1	3	6	10	15	21
1		>0.999	**0.004**	0.071	0.144	0.214
3			**0.011**	0.115	0.206	0.286
6				>0.999	>0.999	>0.999
10					>0.999	>0.999
15						>0.999
21						

Table S7. Statistical analysis results (adjusted p-values) of **biofilm viability** comparisons by means of intact cell membrane over time. Data (at least N = 15 individual images per condition), were tested using 2-way ANOVA with Tukey's multiple comparison test. Family-wise significance level was set to α = 0.05 and statistically significant differences are highlighted in bold.

Static Cultivation

Commensal Model

	1	3	6	10	15	21
1		**0.008**	>0.999	>0.999	0.971	>0.999
3			**0.001**	**0.001**	**0.037**	**0.002**
6				>0.999	0.856	>0.999
10					0.894	>0.999
15						0.955
21						

Dysbiotic Model

	1	3	6	10	15	21
1		**<0.001**	**<0.001**	**<0.001**	**<0.001**	**<0.001**
3			**<0.001**	**<0.001**	**<0.001**	**<0.001**
6				0.388	0.998	0.449
10					0.182	**0.003**
15						0.718
21						

HOBIC Cultivation

Commensal Model

	1	3	6	10	15	21
1		**<0.001**	**<0.001**	**<0.001**	**<0.001**	**<0.001**
3			0.949	**<0.001**	**<0.001**	**<0.001**
6				**<0.001**	**<0.001**	**<0.001**
10					0.980	>0.999
15						>0.999
21						

Dysbiotic Model

	1	3	6	10	15	21
1		0.316	**<0.001**	**<0.001**	**<0.001**	**<0.001**
3			**<0.001**	**<0.001**	**<0.001**	**<0.001**
6				0.841	0.427	0.537
10					0.985	0.996
15						>0.999
21						

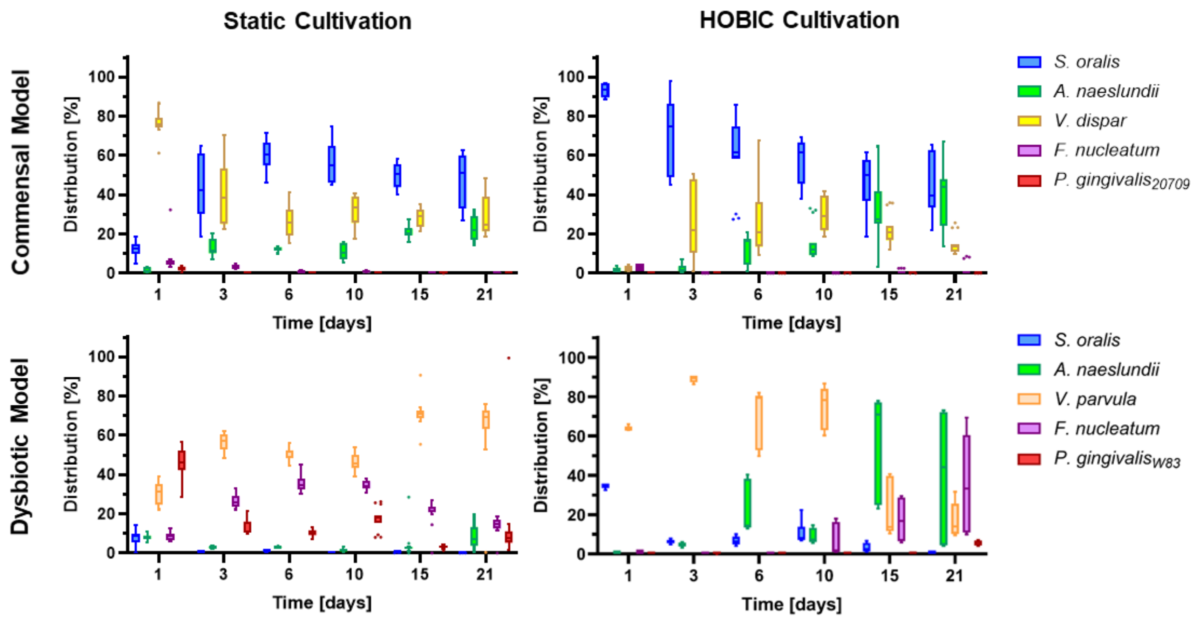

Figure S1. Total bacterial species distribution over time in the different oral multispecies biofilm models. Tukey box plots of individual species distributions of the commensal and dysbiotic models during static and HOBIC cultivation over time quantified by qRT-PCR. Statistical comparisons for individual species development over time (N = 9 replicates per condition) was done using 2-way ANOVA with Dunnett's test for multiple comparison to family-wise α = 0.05. Results are given in Supplementary Table S9.

Table S8. Statistical analysis results (adjusted p-values) of **viable biofilm species distribution** (qRT-PCR with PMA pre-treatment) over time compared to day 1. Data (N = 9), were tested using 2-way ANOVA with Dunnett's multiple comparison test. Family-wise significance level was set to $\alpha = 0.05$ and statistically significant differences are highlighted in bold.

Static Cultivation

Commensal Model

1 vs.	3	6	10	15	21
S. oralis	**<0.001**	**<0.001**	**<0.001**	**<0.001**	**<0.001**
A. naeslundii	0.347	0,494	0,095	**<0.001**	**<0.001**
V. dispar	**<0.001**	**<0.001**	**<0.001**	**<0.001**	**<0.001**
F. nucleatum	0.998	0.991	0.996	0.987	0.986
P. gingivalis	>0.999	>0.999	>0.999	>0.999	>0.999

Dysbiotic Model

1 vs.	3	6	10	15	21
S. oralis	**<0.001**	**<0.001**	**<0.001**	**<0.001**	**<0.001**
A. naeslundii	0.268	0.297	**0.040**	0.999	**0.015**
V. parvula	0.884	0.999	0.908	**<0.001**	**<0.001**
F. nucleatum	**<0.001**	0.092	**0.001**	**<0.001**	**<0.001**
P. gingivalis	0.962	**0.001**	**<0.001**	**<0.001**	**<0.001**

HOBIC Cultivation

Commensal Model

1 vs.	3	6	10	15	21
S. oralis	0.091	0.907	0.079	**<0.001**	**<0.001**
A. naeslundii	0.981	>0.999	0.204	**<0.001**	**<0.001**
V. dispar	0.992	0.291	**0.037**	**0.004**	0.068
F. nucleatum	0.108	0.108	0.108	0.144	0.364
P. gingivalis	>0.999	>0.999	>0.999	>0.999	>0.999

Dysbiotic Model

1 vs.	3	6	10	15	21
S. oralis	0.986	0.989	0.517	0.998	0.853
A. naeslundii	0.300	0.934	0.571	**<0.001**	**<0.001**
V. parvula	0.257	0.489	**0.001**	**<0.001**	**<0.001**
F. nucleatum	0.797	0.779	0.167	**<0.001**	**<0.001**
P. gingivalis	0.953	0.953	0.953	>0.999	**0.012**

Table S9. Statistical analysis results (adjusted p-values) of **total biofilm species distribution** over time compared to day 1. Data (N = 9), were tested using 2-way ANOVA with Dunnett's multiple comparison test. Family-wise significance level was set to α = 0.05 and statistically significant differences are highlighted in bold.

Static Cultivation

Commensal Model

1 vs.	3	6	10	15	21
S. oralis	**< 0.001**	**< 0.001**	**< 0.001**	**< 0.001**	**< 0.001**
A. naeslundii	**< 0.001**	**< 0.001**	**< 0.001**	**< 0.001**	**< 0.001**
V. dispar	**< 0.001**	**< 0.001**	**< 0.001**	**< 0.001**	**< 0.001**
F. nucleatum	0.456	0.067	0.073	**0.037**	**0.036**
P. gingivalis	0.891	0.877	0.876	0.876	0.876

Dysbiotic Model

1 vs.	3	6	10	15	21
S. oralis	**0.015**	**0.028**	**0.008**	**0.009**	**0.007**
A. naeslundii	0.124	0.118	**0.026**	0.381	0.999
V. parvula	**< 0.001**	**< 0.001**	**< 0.001**	**< 0.001**	**< 0.001**
F. nucleatum	**< 0.001**	**< 0.001**	**< 0.001**	**< 0.001**	0.093
P. gingivalis	**< 0.001**	**< 0.001**	**< 0.001**	**< 0.001**	**< 0.001**

HOBIC Cultivation

Commensal Model

1 vs.	3	6	10	15	21
S. oralis	**< 0.001**	**< 0.001**	**< 0.001**	**< 0.001**	**< 0.001**
A. naeslundii	0.998	0.371	**0.036**	**< 0.001**	**< 0.001**
V. dispar	**< 0.001**	**< 0.001**	**< 0.001**	**< 0.001**	0.067
F. nucleatum	0.954	0.954	0.953	0.979	0.999
P. gingivalis	> 0.999	> 0.999	> 0.999	> 0.999	> 0.999

Dysbiotic Model

1 vs.	3	6	10	15	21
S. oralis	**< 0.001**	**< 0.001**	**0.004**	**< 0.001**	**< 0.001**
A. naeslundii	0.928	**< 0.001**	0.501	**< 0.001**	**< 0.001**
V. parvula	**0.002**	0.714	0.434	**< 0.001**	**< 0.001**
F. nucleatum	0.999	> 0.999	0.803	0.081	**< 0.001**
P. gingivalis	> 0.999	> 0.999	> 0.999	> 0.999	0.793

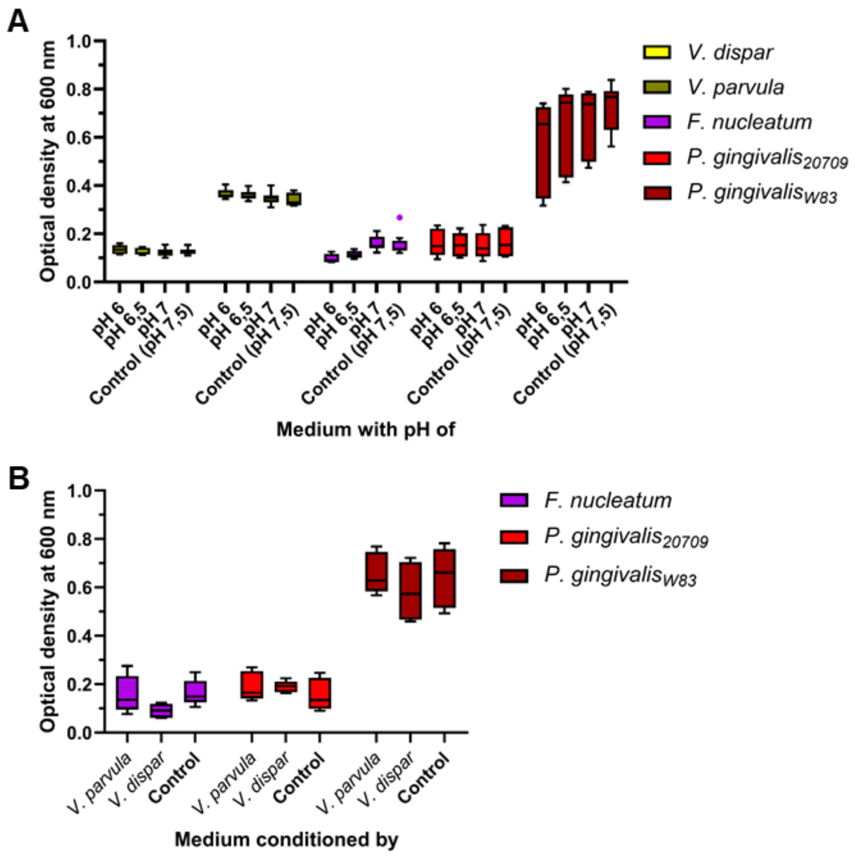

Figure S2. Mean ± standard deviation (N = 9) of bacterial growth under different conditions. (A) Indicated bacterial strains were pre-cultured as described. Cells were harvested and inoculated (final optical density at 600 nm 0.05) into BHI+VitK/Hem medium adjusted to different pH values. After 24 hours of cultivation at 37 °C under anaerobic conditions, bacterial growth was measured using a photometer (BioPhotometer, Eppendorf SE, Hamburg, Germany). (B) Indicated bacterial strains were pre-cultured as described. Veillonella strains were separated from the culture medium by centrifugation and filtration. Fusobacterium and Porphyromonas cells were harvested and inoculated into the different Veillonella-conditioned media (final optical density at 600 nm 0.05) and fresh BHI+VitK/Hem as control medium. After 24 hours of cultivation at 37 °C under anaerobic conditions, bacterial growth was measured using a photometer.
